# Warning from constricted and wrinkled internal jugular vein

**DOI:** 10.1007/s10157-018-1616-9

**Published:** 2018-07-16

**Authors:** Kyohei Ogawa, Keita Hirano

**Affiliations:** Division of Nephrology, Department of Internal Medicine, Ashikaga Red Cross Hospital, 284-1 Yobe-cho, Ashikaga, Tochigi 326-0843 Japan

**Fig. 1:**
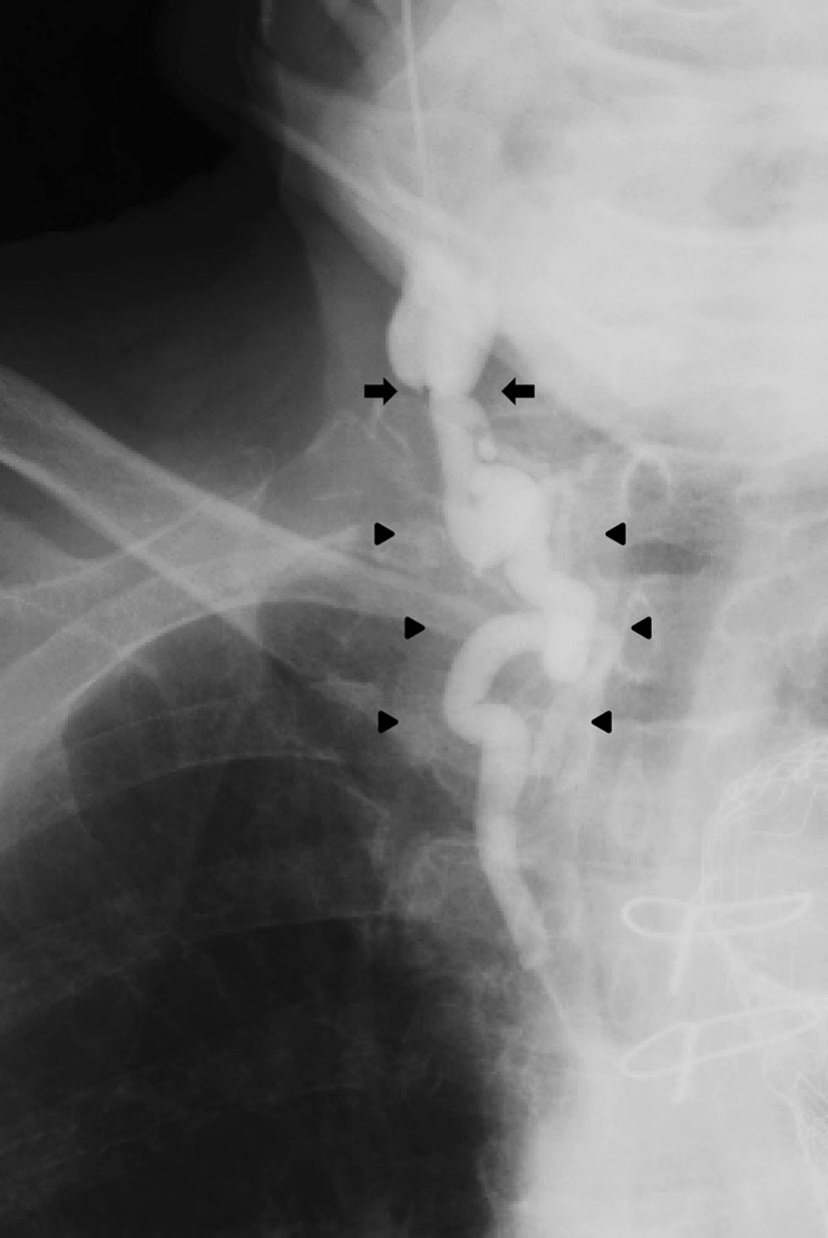
Selective venography via right internal jugular vein. The venography showed narrowing (arrows) and meandering (arrow heads) of the central part of the internal jugular vein, which inhibited guiding wire insertion

A 76-year-old man was admitted to our hospital for induction of hemodialysis due to advanced diabetic nephropathy. Since he had no permanent blood access, insertion of a temporary hemodialysis catheter was attempted at the right internal jugular vein. As the targeted vessel was easily confirmed by ultrasound, the initial puncture was not difficult. However, when the guiding wire was introduced, it did not advance beyond 10 cm from the puncture site. Venography was immediately performed, which showed narrowing and meandering of the central part of the internal jugular vein, which was hardly confirmed by ultrasound (Fig. 1). Finally, another temporary hemodialysis catheter was inserted at the right femoral vein. Today, ultrasound-guided method is the mainstay for internal jugular vein cannulation at bedside [1]. However, the present case demonstrated an important disadvantage of ultrasound-guided cannulation. Physicians should be reminded of clinical implications of classical venography in cases where smooth passage of guiding wire is impossible under ultrasound guidance.
